# Perspectives on Medical Services Integration among Conventional Western, Traditional Korean, and Dual-Licensed Medical Doctors in Korea

**DOI:** 10.1155/2013/105413

**Published:** 2013-12-07

**Authors:** Junghwa Lim, Youngju Yun, Sangyeoup Lee, Younghye Cho, Han Chae

**Affiliations:** ^1^Division of Neuropsychiatry, School of Korean Medicine, Pusan National University, Yangsan, Republic of Korea; ^2^Division of Integrative Medicine, School of Korean Medicine and Medical Research Institute, Pusan National University, Yangsan, Republic of Korea; ^3^Medical Education Unit and Medical Research Institute, Pusan National University School of Medicine, Yangsan, Republic of Korea; ^4^Family Medicine Clinic and Research Institute of Convergence of Biomedical Science and Technology, Pusan National University Yangsan Hospital, Yangsan, Republic of Korea; ^5^Division of Longevity and Biofunctional Medicine, School of Korean Medicine, Pusan National University, Yangsan, Republic of Korea

## Abstract

The aim of this study was to examine the perspectives on the options for the integration of western and traditional Korean medical services among three types of medical doctors with different disciplines in Korea. We surveyed and analyzed responses from 167 conventional Western medicine (WM), 135 traditional Korean medicine (KM), and 103 dual-licensed (DL) doctors who can practice both. All three kinds of doctors shared similar attitude toward license unitarization. KM doctors most strongly agreed on the need of the cooperative practice (CP) between KM and WM and on the possibility of license unitarization among three groups. DL doctors believed that CP is currently impracticable and copractice is more efficient than CP. WM doctors presented the lowest agreement on the need of CP and showed lower expectation for DL doctors as mediators between WM and KM than others. This study showed the difference of perspectives on the options for the integrative medical services among three different doctor groups in Korea. More studies are required to explore the underlying reasons for these discrepancies among WM, KM, and DL doctors.

## 1. Introduction

Since the medical paradigm is shifting from disease-centered biomedicine toward patient-centered care, integrative medicine embracing conventional medicine and complementary and alternative medicine (CAM) has emerged as a new vision of future healthcare system [1]. Asian countries legitimately recognized their own traditional medicine as conventional medicine in healthcare system [2]. China is operating two different medical disciplines within an integrated system under the government's leadership [3], and Taiwan, Hong Kong, and Korea have a parallel medical system in which traditional and conventional western medical doctors coexist within their respective medical systems independently [4, 5].

However, this type of medical system inevitably gives rise to the structural conflicts between medical professionals. The traditional Korean medicine (KM) and KM doctors, which have increasing volume of reimbursements in National Health Insurance and adopted studies with modern biomedicine [6], hold the same legal position with WM doctors as certified medical practitioners. Although medical providers and consumers have strong consensus on the integration of KM and WM as desirable future of Korean medical system [7], turf wars surrounding newly developed procedures and diagnostic examinations have arisen frequently because two groups have to share fixed National Health Insurance reimbursement.

There have been ongoing discussions on some options for dissolving conflicts and integration of two medical services including the cooperative practice (CP) between WM and KM, which is the medical practice that WM and KM doctors participate in together based on mutual respect and shared medical procedure, the license unitarization which is defined in our study as assimilation of WM and KM as a single unified education and license system [8], and copractice by DL doctors who have the right to practice the KM clinical procedures like acupuncture and herbal medicines alongside WM instruments on specific patients [9]. However, none of these options reached the consensus [7, 10, 11].

Since the amendment of the National Medical Act in 1951, KM and WM doctors have coexisted in Korean medical service system [7]. Holliday suggested Korea had the clearest model of balanced healthcare development [12]; however, Korea has suffered more from conflicts between WM and KM doctors than other east Asian countries [13].

To resolve the conflict, facilitating CP has been the state's main healthcare policy, and medical institution implementing CP has increased since the 1990s [11]. However, regarding organizational strategies to facilitate CP suggested in international experiences [14] including leadership by dual trained clinicians, bridge building activities, use of condition-specific referral protocols, shared electronic health records, consensuses have barely reached.

With another method for addressing the complicated healthcare issues in Korea, license unitarization has long been discussed in the medical community since the 1970s; the Korean Medical Association has argued for unification of two separate WM and KM licenses. KM doctors have been rather passive and defensive about license unitarization because under the dominance of WM in Korea, unified medical services would mean diminishment or eventual demise of the distinctive features of KM [7]. However, some KM doctors, particularly young KM doctors working at hospitals, started arguing for unification due to the expectations of economic benefits, obtaining the authority to use modern medical equipment [15].

Meanwhile, the number of DL doctors holding WM and KM licenses altogether has risen sharply since the mid-1990s, and it is expected to grow steadily in the future. The DL doctors have the right to copractice under the amendment of Medical Practice Law. And the DL doctors are expected to contribute to the resolution of conflicts between two medical disciplines and facilitate the cooperation and/or integration of WM and KM [11].

Interdisciplinary medical education between WM and KM has been mentioned as not only a prerequisite for CP but also the measure to improve CP and the integration of medical service [11, 16]. The addition of CAM elements in biomedicine curricula is gaining popularity worldwide [17]. In Korea, in 2010, the number of medical schools offering courses on KM has increased to 35 (85.4%) out of 41 [18], but most WM schools give only 2 to 4 hours of lectures on KM in the whole curriculum. This is a very small amount of time compared to Japanese medical school running 16-hour courses on traditional medicine [19]. Considering the current Korean medical situation, in the near future, it will be of great academic interest to view how the medical professionals reach an agreement regarding the measure in the integration of KM and WM.

Before conducting an investigation with more expanded inclusion of medical professions, we established a preliminarily survey on the perspectives on the options under discussion for integrating medical services in Korea among medical doctors involved in CP.

We supposed that (1) medical professionals working at medical institutions offering CP would share similar opinions regarding the appropriateness of CP and license unitarization, but (2) would present different perspectives concerning the options for integration of medical services among groups. This study would provide a foundation for the better understanding of the present situation and more rigorously designed study of the forthcoming.

## 2. Method

### 2.1. Subjects and Procedures

The DL doctors included in this study were registered members of the Korean Association of the DL doctors. We sent emails to 190 DL doctors and finally got 103 (54.2%) respones for the analysis for 4 weeks from the 6th of January 2011.

We also distributed the questionnaire to 200 WM and 200 KM doctors working at a total of five university hospitals and Korean traditional hospitals selected in consideration of region and duration of offering CP via email and also 100 WM doctors attending CAM-related symposiums in person during 6 weeks from the 20th of June 2011. We totally collected responses from 171 (57%) WM doctors and 135 (67.5%) KM doctors; however, 4 responses from WM doctors with many blanks were excluded for the analysis.

### 2.2. Questionnaire for the Survey

We carefully reviewed previous studies [9, 10, 20] focusing on the attitude of medical doctors toward CAM, CP, and license unitarization for selecting questionnaire items. CP, license unitarization, and copractice were found to be the options for the integration between two medical paradigms in Korea. The questionnaire items were formulated by YJY who performs clinical practice as a DL doctor. The initially developed questionnaires were reviewed and edited by SYL and YHC from western medicine education unit and distributed to the panel of experts for the understanding and response.

The survey questionnaires consist of three sections, 16 items. The first section has questions on the sociodemographic characteristics [9, 21] such as gender, age, acquisition of clinical specialist, and practice setting. The second section has 7 items on the options for integration of medical services. The third section has 5 items on interdisciplinary medical education currently performed in WM school and KM school. Each item is measured using a five-point Likert scale (1 = strongly disagree, 2 = disagree, 3 = neutral, 4 = agree, and 5 = strongly agree).

## 3. Statistical Analysis

Since the distribution of variables did not show normal distribution, Kruskal-Wallis test was used for the examination of differences in responses on the options for integration of medical services between WM, KM, and DL doctors, and Bonferroni method was used for post hoc analysis. Mann-Whitney *U* test was used for detecting the differences in responses on interdisciplinary medical education variables when comparing WM and KM doctor groups to that of the DL doctor group. We divided the DL doctors into WM-based and KM-based DL subgroups depending on which license they have obtained first, and compared the response to examine whether there are differences.

The data are presented as means and standard deviations or frequency with percentage. All analyses were performed using PASW Statistics 18.0 for Windows (IBM, Armonk, NY, USA), and *P* values of 0.05, 0.01, and 0.001 were used for significance.

## 4. Results

### 4.1. Demographic Characteristics

Table 1 shows general characteristics of the participants, in this study. The male portion of the respondents in WM, KM, and DL doctors were 65.9%, 72.6%, and 82.5%, respectively. As for the KM doctors, 37.8% were in their 20s; however, WM (29.3%) and DL (45.6%) doctors were in their 30s.

WM (88.6%) and KM (60.7%) doctors are enrolled in the hospital, and the prevalence of clinical specialist in WM and KM doctor groups were 97.0% and 75.6%. Although 54.4% of the DL doctors practice as general practitioners, KM-based DL doctors reported “working in hospital” (44.2%) rather than the “working in private clinic” (23.2%).

### 4.2. Medical Service Integration in Korea

**Figure 1:**
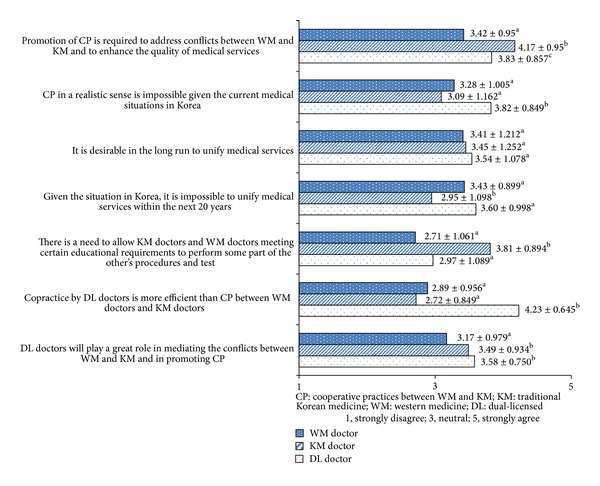
Perception on the options for integration of medical services in Korea.

Perception among three groups on the medical service integration in Korea is presented in Figure 1. There were significant differences among three groups concerning all items except desirability of license unitarization.

Regarding the need for promoting CP, significant differences were founded among three groups (*χ* ^2^ = 57.412, *P* < 0.001). KM doctors presented higher agreement (4.17 ± 0.655) than DL doctors (3.83 ± 0.857), and DL doctors agreed more highly than WM doctors (3.42 ± 0.954).

Regarding the impracticability of CP, it was found that there was significant difference (*χ* ^2^ = 27.709, *P* < 0.001) among groups, DL doctors (3.82 ± 0.849) agreed more highly than the WM doctors (3.28 ± 1.005), and DL doctors presented higher agreement than KM doctors (3.09 ± 1.167). As for the need for license unitarization, there is no statistical differences (*χ* ^2^ = 0.686, *P* = 0.710) among WM (3.41 ± 1.212), KM (3.45 ± 1.252), and DL doctors (3.54 ± 1.078).

Pertaining to the impossibility of license unitarization within the next 20 years, significant difference was founded among groups (*χ* ^2^ = 25.626, *P* < 0.001), WM doctors (3.43 ± 0.899) showed higher agreement than KM (2.59 ± 1.098), and DL doctors (3.60 ± 0.998) expressed higher agreement than KM doctors.

Concerning the need for crossover practice, that is, allowing those medical procedures that have been previously under the other group's purview to be performed, there was significant difference among groups (*χ* ^2^ = 77.807, *P* < 0.001), KM doctors (3.81 ± 0.894) expressed higher agreement compared to WM doctors (2.71 ± 1.061), and DL doctors (2.97 ± 1.089). Concerning efficiency of copractice, significant difference was founded among groups (*χ* ^2^ = 139.142, *P* < 0.001), DL doctors (4.23 ± 0.645) showed higher agreement than WM doctors (2.89 ± 0.956) and KM doctors (2.72 ± 0.904). Regarding the role of DL doctors, there was significant difference among groups (*χ* ^2^ = 15.206, *P* < 0.001), KM doctors (3.49 ± 0.934) and DL doctors (3.58 ± 0.750) showed higher agreement than WM doctors (3.17 ± 0.979).

### 4.3. Interdisciplinary Medical Education

The perceptions among WM doctors and DL doctors about KM education in WM schools are shown in Table 2, and the perceptions among KM doctors and DL doctors about WM education in KM schools are displayed in Table 3.

Both the WM doctors and DL doctors agreed on the need for KME in WM schools. Especially, the DL doctors showed stronger agreement on the need for a quantitative increase statistically than the other group (*P* = 0.003).

Regarding WM education in KM schools, KM doctors and KM-based DL doctors generally did not agree with the statement that WM education takes up excessive portion of KM curriculum (2.74 ± 1.071, 2.61 ± 0.915), while WM-based DL doctors generally agreed with this (3.34 ± 1.044, *P* < 0.0001). KM doctors did not agree with the statement that the amount of WM education is insufficient (2.90 ± 0.936), but agreed with the statement that the quality of education is unsatisfactory (3.52 ± 0.991). Meanwhile, the DL doctors agreed with insufficiency in terms of both quantity (*P* = 0.004) and quality (*P* < 0.0001) more strongly than KM doctors and this tendency was more pronounced among KM-based DL doctors.

## 5. Discussion

The distribution of characteristics of the WM, KM, and DL doctors in this study is similar to the previous studies [11, 22, 23]; meanwhile, hospital employees and medical specialty acquisition constituted a higher percentage than WM and KM doctors at large [24, 25]. Hsiao et al. reported that age, practice setting, and training could be important factors in determining clinicians' orientation of integrative medicine [26]; therefore, we considered these characteristics in analyzing the results.

### 5.1. Perception about Integration of Medical Services

This study suggested that three groups—KM, WM, and DL doctors—involved in CP had different attitudes toward the options for medical service integration including CP, license unitarization, crossover practice, copractice, and the roles of DL doctors, although all three groups had similar perspectives on the desirability of license unitarization.

Respondents' anticipation for medical system combining advantages of two disciplines [7, 27] as well as a change in KM doctors' perception regarding license unitarization in a positive way [28] would be reflected in the similar view about license unitarization among three groups.

KM doctors showed higher agreement on the necessity of fostering CP, crossover practice, and the possibility of license unitarization than others. It may be influenced by KM doctors' hope for acquiring more advantages to perform medical practice through CP, crossover practice, or license unitarization.

Contrary to other countries where WM doctors are often the leaders of CP [29], most CP have been implemented at KM hospitals so far [20]; KM doctors have taken a more active part in CP as shown by previous researches [16, 30, 31]. Also, using WM devices by KM doctors is illegal; KM doctors obtained actual benefits from CP in their medical practice requesting WM hospitals to perform requisite medical examinations [15].

DL doctors presented higher agreement on the impracticability of CP and the efficiency of copractice than others. DL doctors are expected as mediators between two medical disciplines [32] and international experiences suggest that integration can be best led by DL clinicians [14]. However, DL doctors' experiences in the realistic difficulties and limitations of CP [32] would have influenced their skeptical perspective on CP. Moreover, as copractice by DL doctors was made possible from 2009 [9], they may consider copractice as an alternative to CP.

WM doctors showed lower agreement on the necessity of fostering CP and the expectation of DL doctors' role as mediator between two medical paradigms than others. WM doctors working at hospital offering CP have a more favorable attitude toward CP in previous researches [16, 30, 31]; however, a recent study [10] reported the decrease of positive perception on the need for CP in WM doctors. It is thought that this trend had an effect on the lowest agreement in WM doctors.

The number of KM-based DL doctors has increased sharply since 2010, and a substantial number of them are working at hospitals [11]. These streams may have influenced higher agreement among the KM doctors regarding DL doctors' role as mediators.

Though taking social contextual differences into account, some results of this study can be interpreted in the context of wider healthcare policies. Similar to our results, opinion disagreement on medical services integration between medical professionals was found in policy developing research of Hong Kong [33] especially in the area of crossover practice.

The subjects' responses reflect each group's interests, hopes, and anticipation rather than being an objective evaluation or prediction, and we did not overlook this when analyzing study results. The subtext of disagreements among all kind of doctors should be investigated by future studies.

### 5.2. Perception about Interdisciplinary Medical Education

DL doctors showed higher agreement on the need for KM education and quantitative growth than WM doctors. One systematic review study reported similar result that WM doctors and medical students had more positive attitude and perceptions and improved knowledge after CAM educations. In particular WM doctors trained in CAM are often better accepted than other medical professionals trained only in CAM [17].

Meanwhile, DL doctors more strongly agreed with the excessiveness of WM education portion and insufficiency of quantity and quality of WM education in KM schools. Particularly, WM-based DL doctors showed stronger perception on the excessively high proportion of WM education in KM school compared to KM-based DL doctors.

We thought that perspectives on quantity and quality of WM education in KM schools among the DL doctors (especially KM-based DL doctors) imply the need for improvement of WM education in KM schools.

In Korea, there have not been special license examinations or integrated education unlike Taiwan and China, so the candidates for DL doctors are required to attend both (WM and KM) medical schools [28]. Therefore, the opinions of DL doctors are important to seek the improvement plan for interdisciplinary medical education at colleges.

Further survey and in-depth interviews on the reasons of the unsatisfaction of WM education in KM schools and the measures for improvement are required.

### 5.3. Significance and Limitation of the Research

This research is significant in the sense that it is the first-ever study to investigate the perception about medical service integration among all concerned parties—WM, KM, and DL doctors—in Korea. Also, we believe that this study has significance to involve a considerable number of DL doctors which is almost half of the entire DL doctors, reported to be 206 in 2010 [11].

This study is limited in the following aspects: first, the study subject groups, WM and KM doctors, were exclusively confined to those who work at medical institutions offering CP and those who are interested in CP and thus may not fully represent the perception among the overall WM and KM doctors. Nevertheless, this study aimed to compare and confirm the differences of perspectives on medical service integration among medical practitioners involved in CP, since their experience-based insight would be important for methodological development of medical service integration. Therefore, we determined that the aforementioned study participants were suitable subjects for this study.

Second, items included in the survey questionnaire for this study could be used only to confirm the overall consensus and difference in perception among different groups regarding medical service integration as well as interdisciplinary medical education at colleges but failed to explain the specific reasons behind the participants' perception. Further qualitative research should follow to find out the reasons why three groups were divided over most of the issue. Similarly, health policy research using focus groups and Delphi technique can help three kinds of medical practitioners to arrive at common strategies for medical service integration, that is, agreeable to all three parties.

## 6. Conclusion

The conflicts between two medical disciplines are grave in Korea as compared with other east Asian countries due to its unique dual medical system. So, some options such as CP, license unitarization, and copractice are suggested to integrate two medical services.

Our finding supported the hypothesis that the perception on the options for medical service integration would be divided among three medical professionals including WM, KM, and DL doctors involved in CP, although all three groups agreed on the need for license unitarization. We thought these split perspectives would reflect the desires and hopes of three medical professionals and this perception gap is expected to be even greater among the entire WM, KM, and DL doctors. The reasons inherent in discrepancies should be explored via future researches.

## Figures and Tables

**Table 1:** General characteristics of the subjects in this study.

	WM doctor (*n* = 167)	KM doctor (*n* = 135)	DL doctor		
			Total (*n* = 103)	WM-based (*n* = 60)	KM-based (*n* = 43)
Sex					
Male	110 (65.9)	98 (72.6)	85 (82.5)	51 (85.0)	34 (79.1)
Female	56 (33.5)	37 (27.4)	18 (17.5)	9 (15.0)	9 (20.9)
n.a.	1 (0.6)				
Age					
Over 50	36 (21.6)	14 (10.4)	12 (11.6)	7 (11.7)	5 (11.7)
41–50	45 (26.9)	31 (23.0)	33 (32.0)	25 (41.7)	8 (18.6)
31–40	49 (29.3)	39 (28.9)	47 (45.6)	27 (45.0)	20 (46.5)
21–30	36 (21.6)	51 (37.8)	11 (10.7)	1 (1.7)	10 (23.3)
n.a.	1 (0.6)				
Clinical specialist					
Yes	162 (97.0)	102 (75.6)	42 (40.8)	18 (29.0)	24 (55.8)
WM specialist	162 (97.0)		37 (35.9)	16 (26.7)	21 (48.8)
KM specialist		102 (75.6)	7 (6.8)	2 (3.3)	5 (11.6)
Both			2 (1.9)		2 (4.7)
No	5 (3.0)	33 (24.4)	59 (57.3)	41 (68.3)	18 (41.8)
n.a.			2 (1.9)	1 (1.7)	1 (2.4)
Practice settings					
Local clinic	17 (10.2)	53 (39.3)	56 (54.4)	46 (76.6)	10 (23.2)
Hospital	148 (88.6)	82 (60.7)	31 (30.1)	12 (20.0)	19 (44.2)
Others			13 (12.6)	1 (1.7)	12 (27.9)
n.a.	2 (1.2)		3 (2.9)	1 (1.7)	2 (4.7)

Data shown as frequency (%).

WM: western medicine; KM: traditional Korean medicine; DL: dual-licensed; n.a.: not available.

**Table 2:** Perception on the KM education in WM school.

Questionnaire items	WM doctor	DL doctor		WM-based DL doctor	KM-based DL doctor	
It is unnecessary to learn KM in WM schools	2.57 ± 0.931	2.24 ± 0.902	*Z* = −3.237**	2.27 ± 0.954	2.21 ± 0.833	*Z* = −0.127
It is required to increase the amount of KM education in WM schools	3.28 ± 0.996	3.65 ± 0.987	*Z* = −2.937**	3.68 ± 1.017	3.60 ± 0.955	*Z* = −0.579

**P < 0.01.

KM: traditional Korean medicine; WM: western medicine.

**Table 3:** Perception on the WM education in KM school.

Questionnaire items	KM doctor	DL doctor		WM-based DL doctor	KM-based DL doctor	
WM education takes up excessive portion of curriculum in KM schools	2.61 ± 0.915	3.09 ± 1.091	*Z* = −3.519***	3.34 ± 1.044	2.74 ± 1.071	*Z* = −2.725**
The amount of WM education allocated to KM schools is insufficient	2.90 ± 0.936	3.26 ± 1.024	*Z* = −2.918**	3.19 ± 0.991	3.37 ± 1.070	*Z* = −1.011
The quality of WM education in KM schools is unsatisfactory	3.52 ± 0.991	4.04 ± 0.900	*Z* = −4.170***	3.90 ± 0.923	4.23 ± 0.841	*Z* = −1.946

**P < 0.01; ***P < 0.001.

KM: traditional Korean medicine; WM: western medicine.

## Appendices

### A. General Characteristic

1. What is your sex?
  □ male
  □ female
2. When are you born?
  □ before 1950
  □ 1951–1960
  □ 1961–1970
  □ 1971–1980
  □ after 1981
3. Did you acquire clinical specialist or are you expected to?
  □ Yes
  □ No
4. Where is your current work place?
  □ WM hospital
  □ KM hospital
  □ WM private clinic
  □ KM private clinic

### B. Integration of Medical Services

See Figure 1.

### C. Interdisciplinary Medical Education

See Tables 2 and 3.
